# Objective Physical Activity Accumulation in Bouts and Nonbouts and Relation to Markers of Obesity in US Adults

**Published:** 2008-09-15

**Authors:** Scott J Strath, Robert G Holleman, Caroline R Richardson, David L Ronis, Ann M Swartz

**Affiliations:** Department of Human Movement Sciences, The University of Wisconsin-Milwaukee; Department of Family Medicine, University of Michigan Medical School, and Health Services Research and Development, US Department of Veterans Affairs Medical Center, Ann Arbor, Michigan; Department of Family Medicine, University of Michigan Medical School, and Health Services Research and Development, US Department of Veterans Affairs Medical Center, Ann Arbor, Michigan; School of Nursing, University of Michigan, and Department of Family Medicine, University of Michigan Medical School, Ann Arbor, Michigan; Department of Human Movement Sciences, University of Wisconsin-Milwaukee, Milwaukee, Wisconsin

## Abstract

**Introduction:**

Little is known about the relation between duration of physical activity and obesity. The objective of this study was to compare the effects of physical activity in bouts (≥10 minutes) to the effects of physical activity in nonbouts (<10 minutes) on markers of obesity.

**Methods:**

We used data from the 2003-2004 National Health and Nutrition Examination Survey on body mass index, waist circumference, and objectively determined physical activity levels for 3,250 adults aged 18 years or older. After controlling for relevant confounding variables, we performed multiple linear regression analyses to predict body mass index and waist circumference for bout and nonbout minutes of moderate- to vigorous-intensity physical activity (MVPA) and for bout and nonbout accelerometer counts of physical activity.

**Results:**

MVPA bout minutes and MVPA nonbout minutes are independently associated with body mass index and waist circumference, after controlling for confounding variables. The strength of the association between lower body mass index and MVPA bout minutes (β = −0.04, *P* <.001) was nearly 4 times greater than for MVPA nonbout minutes (β = −0.01, *P* = .06). For smaller waist circumference the association was nearly 3 times greater for MVPA bout minutes (β = −0.09, *P* <.001) than for MVPA nonbout minutes (β = −0.03, *P* = .01). Bout minutes of physical activity were at a higher intensity of activity compared with nonbout minutes of physical activity.

**Conclusion:**

Accumulating MVPA in nonbouts may be a beneficial starting point for individuals to increase physical activity levels and decrease body mass index and waist circumference. However, bouts of physical activity lasting ≥10 minutes may be a more time-efficient strategy to decrease body mass index and waist circumference.

## Introduction

The prevalence of obesity has reached alarming levels in the United States. Results from the 2003-2004 National Health and Nutrition Examination Survey (NHANES) indicate that approximately 65% of US adults are now either overweight or obese (1). This high prevalence raises public health concern because many other chronic health conditions — including hypertension, type 2 diabetes, dyslipidemia, coronary heart disease, and stroke — are associated with elevated body mass index (BMI) (2,3).

Evidence for the health benefits of regular physical activity (PA) has been mounting during the last 3 decades. Regular PA is a key behavioral component in the treatment and prevention of overweight and obesity (4-6). Furthermore, regular PA is one of the best predictors of weight loss maintenance (7). Although the health benefits of regular PA have been clearly recognized and widely promoted, an estimated 55% of US adults are not meeting recommended levels of PA during nonworking hours (8). Lack of time is a frequently cited barrier to regular PA (9). Because of this, accumulating daily doses of moderate-intensity PA, such as in several 8- to 10-minute bouts accumulated throughout the day, has been recommended as an alternative to 1 continuous longer bout of PA (10,11). Shorter bouts of PA may be more easily incorporated into an individual's day than a single, prolonged bout.

A parallel concept to the PA public health recommendations was the notion of an active lifestyle (eg, taking the stairs rather than the elevator, parking the car a little farther from your destination). Sessions of lifestyle activity may be as short as 1 to 3 minutes in duration. Although engaging in these brief activities is commonly recommended, there is little evidence that these short episodes of activity improve health.

To accurately investigate the relationship between health parameters and "nonbouts" of PA (activity of <10 minutes in duration), an objective PA measurement tool able to capture intermittent lifestyle activity behaviors is necessary. The 2003-2004 NHANES survey included detailed objective assessment of PA using accelerometers and objective measures of obesity in a large nationally representative sample of Americans. We used these data to compare the effect of bouts of PA (activity ≥10 minutes in duration) with that of nonbout activity on markers of obesity in this population-based sample.

## Methods

### Study sample and procedures

NHANES is a cross-sectional observational study conducted by the Centers for Disease Control and Prevention that uses a stratified, multistage probability design to obtain a nationally representative sample of the US population (12). The survey population included clusters of households in the United States. The 2003-2004 NHANES sampled 12,761 people, with 10,122 participants agreeing to interviews and 9,643 participating in physical examinations. The survey consisted of an interview, examination, and laboratory component. A trained interviewer implemented the household interview survey, which assessed numerous variables with self-reported measures.

For our analyses, we included NHANES participants aged ≥18 years, who were not pregnant, had a BMI of ≥18.5 kg/m^2^, and who wore an Actigraph (ActiGraph LLC, Fort Walton Beach, Florida) accelerometer (ACC) to objectively measure PA.

### Measures

BMI (kg/m^2^) was calculated from measured height (m) and mass (kg) obtained at the medical examination centers. Height was measured by using a wall-mounted stadiometer. The stadiometer was calibrated at the start of each week by placing a known height (80 cm) calibration rod up against the horizontal stadiometer bar. Weight was measured with a digital floor scale. The digital floor scale was calibrated at the start of the day by a trained technician with six 50-lb weights, with an acceptable range of precision from 299.75 to 300.25 lbs. Both height and weight data were captured and entered into an electronic database automatically. Standardized procedures were used for both height and weight measurements (13).

Waist circumference measurements were also carried out at the medical examination centers. A steel measuring tape was placed around the upper border of the ilium with measurements taken to the nearest 0.1 cm. Standardized procedures were used for all waist circumference measurements (13).

PA was monitored by the Actigraph AM-7164 ACC. The Actigraph ACC is a lightweight (42 g) and small (5.08 × 4.06 × 1.53 cm) uniaxial ACC powered by a lithium battery. It records accelerations from 0.05 to 2.00 G with frequencies of 0.25 to 2.5 Hz (14). The Actigraph ACC has an internal time clock and extended memory and is able to record and store the magnitude of acceleration and deceleration associated with movement. The recorded signal is then amplified and filtered, and the result is a signal that is scored as an "ACC count." This count can be summed over a user-specified time interval, or epoch. Sixty-second epochs were used by NHANES (15). The monitors were attached to an elastic belt and worn at the right hip by people aged ≥6 years who did not have impairments to walking or wearing the monitor. Individuals wore the monitor for 7 consecutive days. Of the total participants, 6,758 completed the objective PA monitoring portion of the examination.

NHANES used standardized data quality procedures to assess validity and reliability of the Actigraph ACC data (15). We used data considered both valid and reliable according to these procedures for our analysis. Any block of time ≥60 minutes when the ACC count was 0 was considered time when the monitor was not worn. We counted as valid days of data only those days during which participants wore the ACC for at least 600 minutes. Although participants were asked to wear the ACC for 7 consecutive days, some participants did not wear the ACC for the full week. Only participants who had at least 4 days of valid ACC data were included in this analysis.

We coded 1 minute of ACC data as 1 moderate- to vigorous-intensity PA (MVPA) minute if it had an ACC count of ≥760 (16). We defined a bout as ≥10 consecutive minutes of MVPA. From these data, we calculated average daily minutes of MVPA in bouts and nonbouts. All ACC counts in the MVPA range were summed into either MVPA bout activity or MVPA nonbout activity.

### Confounding variables

Potential confounding variables were demographic variables (ie, age, race/ethnicity, smoking status, sex, and self-reported health status)Potential confounding variables were demographic variables (ie, age, race/ethnicity, smoking status, sex, and self-reported health status). We calculated age at time of survey from the participant's self-reported date of birth. All individuals aged ≥85 years were coded by NHANES to be 85 to protect confidentiality. Participants were classified into 1 of 4 categories for race/ethnicity based on self-reported background: white, black, Mexican American, or other. To determine health status we used a single question asking participants to describe their health as poor, fair, good, very good, or excellent, which we converted into a dichotomous variable: 1) those reporting poor or fair health; and 2) those reporting good, very good, or excellent health. Smoking status was determined by measuring plasma serum levels of cotinine, which is a major metabolite of nicotine. A participant was considered a current smoker if he or she had ≥10 ng/dL of cotinine in his or her plasma serum (17).

### Statistical analysis

We performed statistical analyses by using Intercooled Stata 9.2 for Windows (StataCorp LP, College Station, Texas). Descriptive statistics were used for demographic variables, BMI, waist circumference, and PA measures. Participant age, BMI, waist circumference, and PA were normally distributed, and means and standard deviations for these variables were reported. Frequencies of sex, race/ethnicity, smoking status, self-reported health status, and BMI category were also reported. Each measure of PA was stratified by demographic characteristics, and means and standard deviations were reported. We performed 2 different sets of multiple linear regression analyses, 1 using BMI as the dependent variable and 1 using waist circumference as the dependent variable. Two different pairs of predictor variables were used in the multiple regressions. First, we used bout and nonbout minutes of MVPA, and then we used bout and nonbout ACC counts as measures of PA. Appropriate confounders were included, and an age-squared variable was included to account for the nonlinear association between age, BMI, and waist circumference. NHANES uses a complex probability sample with stratification and an unequal probability of selection; therefore, we had to take into account the stratification, clustering, and selection probability of the people in the sample (18). All our analyses used the sampling weight, stratification, and clustering (primary sampling unit) variables.

## Results

The entire NHANES sample of 10,122 participants was reduced as shown in the Figure to contain only those who met the inclusion criteria for the regression analysis for waist circumference (N = 3,272) and for the regression analysis for BMI (N = 3,250). The characteristics of these resulting samples along with sex-specific demographics are shown in Table 1. This sample was older, had a smaller smoking prevalence, and had a lower percentage of racial minorities than those without valid ACC data. Men were considerably more active than were women in both bouts and nonbouts of PA.

**Figure.**
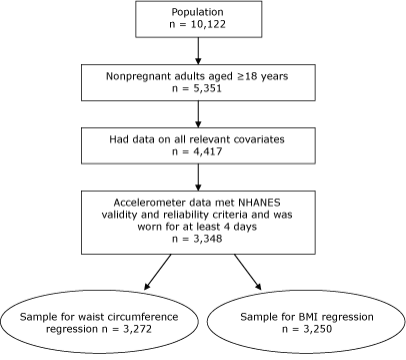
Participants from the National Health and Nutrition Examination Survey Selected for Study of Bouts^a^ and Nonbouts of Physical Activity, 2003–2004 Abbreviation: BMI, body mass index. ^a^ At least 10 consecutive minutes of moderate- to vigorous-intensity physical activity.

Table 2 shows a breakdown of PA by minutes of activity per day and measured ACC counts per day, by demographic characteristics. Most activity consisted of nonbouts of PA. By race/ethnicity, the most active group was Mexican Americans, followed by marginal separations between blacks, whites, and others. Smokers tended to get more PA in nonbouts than nonsmokers, while nonsmokers did slightly more PA in bouts than did smokers. PA in both bouts and nonbouts decreased as age increased. Participants who considered themselves in excellent, very good, and good health were more likely to be physically active than were those who considered themselves in fair or poor health. As the BMI category increased from normal to obese, the activity levels decreased in all groups.

The regression analysis for BMI using bout and nonbout minutes of MVPA per day is shown in Table 3. Adjusting for all confounders, the strength of the association with decreased BMI was nearly 4 times greater for MVPA bout minutes (β = −0.04, *P* <0.001) than for MVPA nonbout minutes (β = −0.01, *P* = .07). The results are similar in the regression analysis for waist circumference, also shown in Table 3. Adjusting for all confounders, the strength of the association with decreased waist circumference was approximately 3 times greater for MVPA bout minutes (β = −0.09, *P* <.001) than for MVPA nonbout minutes (β = −0.03, *P* = .01). We found a significant difference between MVPA bout minutes and MVPA nonbout minutes for both BMI and waist circumference regression analyses. The difference in importance of MVPA bout minutes and MVPA nonbout minutes was greater for women than for men.

The regression analyses for BMI and waist circumference using ACC counts from MVPA bouts and MVPA nonbouts are shown in Table 4. After adjusting for all confounding variables in the total sample, there was a significant difference between MVPA bout ACC counts and MVPA nonbout ACC counts for predicting BMI (β = −0.15, *P* <.001 vs β = −0.07, *P* = .005, respectively) and for predicting waist circumference (β = −0.36, *P* <.001 vs β = −0.23, *P* <.001, respectively). There were, however, statistically significant differences favoring the importance of bout activities among women.

The regression analyses revealed that people who accumulated 30 minutes of MVPA in nonbouts had a BMI that was 0.3 kg/m^2^ lower (β = −0.01 multiplied by 30) and a waist circumference that was 0.9 cm smaller (β = −0.03 multiplied by 30) than the BMI and waist circumference among all other people. People who accumulated 30 minutes of MVPA in bouts lasting 10 minutes or more had a BMI that was 1.2 kg/m^2^ lower (β = −0.04 multiplied by 30) and a waist circumference that was 2.7 cm smaller (β = −0.09 multiplied by 30) than the BMI and waist circumference among all other people. Although all minutes of MVPA met the minimum intensity criteria for MVPA, the mean weighted ACC count in nonbout minutes was considerably lower (1,420 ACC counts per minute) than the mean weighted ACC count in bout minutes (2,370 ACC counts per minute) (data not shown).

## Discussion

PA has many health benefits, and sedentary behaviors are associated with increased risk of many chronic diseases, including obesity and obesity-related diseases and decreased longevity (10,11,19). Recent increases in obesity have been partly attributed to declining PA levels (20). Although there is a consensus on the importance of PA to markers of health such as obesity, the actual amount of PA necessary for health promotion is less clear (21).

PA recommendations have evolved from the amount of exercise required to improve or maintain cardiorespiratory fitness (22) to a public health paradigm recommending that "every U.S. adult should accumulate 30 minutes or more of moderate intensity PA on most, preferably all, days of the week" (10, p. 404). Irrespective of the uncertainty of the PA dose needed to promote health, the fact remains that as a society we practice sedentary behaviors. In an attempt to exchange sedentary behaviors for active ones, public health and clinical recommendations often start with the idea of parking farther from your destination or taking the stairs rather than the elevator. This advice implies that such active lifestyle choices, which may last <10 minutes, are of benefit to health. To our knowledge, no other nationally representative study has examined whether this strategy of accumulating PA in sessions lasting less than 10 minutes is as effective as PA accumulated in bouts lasting 10 minutes or more with relation to markers of obesity.

The principal finding from this study showed that accumulating MVPA in sessions lasting less than 10 minutes (nonbouts) was independently associated with lower BMI and smaller waist circumference even after controlling for other confounding variables, including MVPA accumulated in sessions of 10 minutes or more (bouts). Therefore, accumulating MVPA in nonbouts may be a beneficial starting point to increase PA levels and to lower BMI and decrease waist circumference. Bouts of MVPA had a significantly stronger association with both lower BMI and smaller waist circumference than MVPA in nonbouts. The stronger association reported for bout activity may be due to the greater average intensity of activity during bout versus nonbout minutes. People may essentially work harder during bout minutes than during nonbout minutes spread throughout the day. Thus, people accumulating MVPA in nonbouts throughout the day should probably work toward accumulating PA in bouts throughout the day, because this strategy was more time-efficient in decreasing BMI and waist circumference than was accumulating MVPA in nonbouts throughout the day.

After accounting for confounding variables, PA levels appeared more predictive of waist circumference than did BMI. Waist circumference and BMI are both markers of obesity. However, BMI provides an indicator of overall adiposity and waist circumference provides an indicator of abdominal fat. In addition, waist circumference is not greatly influenced by age, sex, height, or degree of overall adiposity, whereas the ability of BMI to predict overall adiposity is affected by age, ethnicity, body build, and frame size (23-25). This difference could explain why PA appears more predictive of waist circumference than does BMI. Other studies have found similar results. In a randomly selected sample of adult men and women from the AusDiab study, accumulated daily steps were highly predictive of waist circumference (26). Accumulated daily steps predicted BMI in men but not in women (26). Other studies have found stronger relations between objectively determined PA and waist circumference than between objectively determined PA and BMI (27,28).

Previous epidemiologic studies have investigated the effect of duration of exercise on health markers without using objective measures of PA. In the Harvard Alumni Cohort, participants were asked to recall walking, stair climbing, and participation in sports and recreational activities, while noting the frequency and duration of each sport and recreational activity (29). After accounting for confounding variables, the duration of self-reported exercise did not have an independent effect on coronary heart disease risk.

Other intervention studies have compared the effects of accumulated bouts lasting ≥10 minutes to a single continuous bout of PA (ie, 2-3 bouts for 10 minutes compared with a continuous 30-minute bout of activity) (30-40). This literature appears inconclusive. One intervention study reports similar improvements in BMI for the multiple short-bout groups and the single long-bout group (33). Others report that only the longer (continuous 30-minute) exercise sessions were effective in improving markers of obesity (38). Jakicic et al reported in a weight loss and weight maintenance comparison between 1 continuous bout of exercise and multiple short bouts of exercise that no significant differences between groups emerged in BMI or waist circumference after the 18-month intervention (37). In contrast, Donnelly and colleagues compared the effects on BMI of 1 continuous 30-minute exercise session with 2 intermittent 15-minute exercise sessions (35). After completion of the 18-month intervention, they found significant reductions in BMI in the continuous 30-minute exercise session group but observed no change in BMI for the intermittent exercise group (35).

Our study has limitations. It is a cross-sectional analysis, so cause and effect cannot be determined. It is, however, representative of the national population, using strong objective measures of PA and measured markers of obesity. In the literature on methods of PA assessment, the most appropriate regression approach to demarcate PA intensity levels is much debated. We examined both time spent in MVPA, using published regression standards that accommodate both lifestyle and laboratory walking regressions to delineate PA intensity (16), and the sum and average of raw ACC counts. From the methods we used, we could not delineate between different PA domains that would be informative to investigate (eg, the predictive capabilities of transportation activity vs household activity).

The accumulation of PA in nonbouts, a complimentary active lifestyle approach to accumulating PA in bouts, is predictive of lower levels of obesity markers. The concept of activity accumulation appears justified and represents a good stepping stone in getting inactive people to become more active. This concept should provide a strong impetus to sedentary people. However, activity accumulated in bouts lasting ≥10 minutes appears more predictive of lower levels of obesity markers, because a person's activity tends to be at a higher intensity when he or she participates in a sustained bout of PA. Accumulating activity in bouts lasting 10 minutes or more, corresponding to national public health recommendations, is potentially more effective for health promotion as indicated by its stronger predictive relation to obesity markers.

## Figures and Tables

**Table 1:** Characteristics of Selected Participants From the National Health and Nutrition Examination Survey, 2003–2004

**Variable**	**Total (N = 3,272)**	**Men (n = 1,678)**	**Women (n = 1,594)**
**Mean (SD) age, y**	47.2 (17.0)	46.2 (16.8)	48.1 (17.1)
**Mean (SD) BMI^a^, kg/m^2^**	28.3 (6.0)	28.2 (5.1)	28.4 (6.8)
**Mean (SD) waist circumference^b^, cm**	97.1 (15.1)	100.4 (14.1)	93.9 (15.4)
**Mean (SD) bout^c^ MVPA/day, minutes**	14.9 (22.9)	19.1 (26.7)	10.8 (17.5)
**Mean (SD) nonbout MVPA/day, minutes**	90.2 (48.7)	102.7 (53.1)	78.0 (40.4)
**Mean (SD) ACC count/min^d^ during MVPA bout**	2,370 (1,040)	2,370 (1,020)	2,360 (1,060)
**Mean (SD) ACC count/min during MVPA nonbout**	1,420 (230)	1,490 (250)	1,350 (200)
**Mean (SD) bout MVPA ACC counts/day (in thousands)**	39.3 (65.8)	49.5 (75.7)	29.5 (52.7)
**Mean (SD) nonbout MVPA ACC counts/day (in thousands)**	132.4 (80.8)	157.5 (90.1)	108.1 (61.7)
**Sex, %**			
Male	49.2	—	—
Female	50.8	—	—
**Race/ethnicity, %**			
White	75.2	75.0	74.9
Black	9.1	8.8	9.9
Mexican American	10.7	11.2	10.4
Other	5.0	5.1	4.8
**BMI category, %**			
Normal (18.5-24.9 kg/m^2^)	34.0	28.2	37.1
Overweight (25.0-29.9 kg/m^2^)	35.2	41.3	30.3
Obese (≥30 kg/m^2^)	30.8	30.6	32.6
**Smoking status^e^, %**			
Nonsmoker	73.4	66.5	80.5
Smoker	26.7	33.5	19.5
**Self-reported health status, %**			
Excellent, very good, or good	83.7	85.3	81.7
Fair or poor	16.3	14.7	18.3

Abbreviations: BMI, body mass index; MVPA, moderate- to vigorous-intensity physical activity; ACC, accelerometer.

a N = 3,250.

b N = 3,272.

c At least 10 consecutive minutes of MVPA.

d ACC count was derived by using physical activity measurements from the Actigraph AM-7164 activity monitor (ActiGraph LLC, Fort Walton Beach, Florida). Participants wore the monitor, which used an internal time clock and extended memory to record and store the magnitude of acceleration and deceleration associated with movement. The recorded signal was then amplified and filtered, and the resulting signal was scored as an ACC count. One minute of ACC data was coded as 1 minute of MVPA if the ACC count was ≥760 (16).

e Smoking status was determined by measuring plasma serum levels of cotinine, which is a major metabolite of nicotine. A participant was considered a current smoker if he or she had a level ≥10 ng/dL of cotinine in plasma serum (17).

**Table 2:** MVPA Duration and Activity Levels by Demographic Characteristics of Selected Participants From the National Health and Nutrition Examination Survey, 2003-2004

Variable	Bout^a^ MVPA Minutes/Day, Mean (SD)^b^	Nonbout MVPA Minutes/Day, Mean (SD)^b^	Bout^a^ ACC Counts/Day, Mean (SD)^b^	Nonbout ACC Counts/Day, Mean (SD)^b^
**Race/ethnicity**				
White	13.7 (18.9)	87.9 (47.8)	37.4 (61.5)	128.1 (78.8)
Black	15.8 (26.9)	90.6 (46.7)	41.8 (78.4)	134.1 (79.9)
Mexican American	23.6 (38.5)	110.8 (54.5)	54.6 (84.3)	168.6 (91.9)
Other	12.2 (19.8)	79.4 (40.8)	31.4 (52.6)	116.5 (66.3)
**Smoking status^c^**				
Nonsmoker	15.5 (24.1)	87.7 (47.5)	42.1 (70.2)	128.1 (78.6)
Smoker	13.2 (18.9)	97.1 (51.1)	31.9 (51.2)	144.1 (85.5)
**Age, y**				
18-39	19.1 (26.9)	109.1 (48.2)	52.0 (72.8)	169.8 (82.3)
40-59	14.9 (22.0)	95.3 (43.5)	39.2 (67.4)	137.5 (71.3)
≥60	9.0 (15.6)	54.5 (37.3)	21.4 (45.2)	70.4 (52.1)
**Self-reported health status**				
Excellent, very good, or good	15.9 (23.1)	93.4 (47.8)	42.8 (67.7)	138.0 (80.3)
Fair or poor	9.7 (21.1)	73.7 (50.0)	21.7 (51.3)	103.2 (77.3)
**BMI category**				
Normal (18.5-24.9 kg/m^2^)	18.0 (26.7)	93.6 (50.0)	51.2 (75.7)	141.6 (84.8)
Overweight (25.0-29.9 kg/m^2^)	16.0 (22.9)	91.2 (49.5)	42.1 (68.8)	133.6 (82.5)
Obese (≥30.0 kg/m^2^)	10.3 (16.8)	85.4 (45.8)	23.3 (43.9)	121.2 (72.7)

Abbreviations: MVPA, moderate- to vigorous-intensity physical activity; ACC, accelerometer; BMI, body mass index.

a At least 10 consecutive minutes of MVPA.

b ACC count was derived by using physical activity measurements from the Actigraph AM-7164 activity monitor (ActiGraph LLC, Fort Walton Beach, Florida). Participants wore the monitor, which used an internal time clock and extended memory to record and store the magnitude of acceleration and deceleration associated with movement. The recorded signal was then amplified and filtered, and the resulting signal was scored as an ACC count. We coded 1 minute of ACC count data as 1 minute of MVPA if the ACC count was ≥760 (16). ACC counts were divided by 1,000 and then rounded to the nearest hundred.

c Smoking status was determined by measuring plasma serum levels of cotinine, which is a major metabolite of nicotine. A participant was considered a current smoker if he or she had a level ≥10 ng/dL of cotinine in plasma serum (17).

**Table 3:** Reduction in BMI and Waist Circumference by Bout^a^ and Nonbout MVPA Minutes Per Day Among Selected Participants From the National Health and Nutrition Examination Survey, 2003-2004^b^

Category	**Total**		**Men**		**Women**	

	**β Coefficient**	***P *Value**	**β Coefficient**	***P *Value**	**β Coefficient**	***P *Value**
**BMI**						
MVPA bout min/day	−0.04^c^	<.001^d^	−0.02	.002^d^	−0.07^c^	<.001^d^
MVPA nonbout min/day	−0.01	.07	−0.01	.029^d^	−0.01	.42
*R* ^2^	0.08		0.07		0.11	
**Waist circumference**						
MVPA bout min/day	−0.09^c^	<.001^d^	−0.06	.001^d^	−0.15^c^	<.001^d^
MVPA nonbout min/day	−0.03	.01^d^	−0.03	.003^d^	−0.03	.17
*R* ^2^	0.18		0.17		0.15	

Abbreviations: BMI, body mass index; MVPA, moderate- to vigorous-intensity physical activity.

a At least 10 consecutive minutes of MVPA.

b Total and sex-specific models adjusted for age, age-squared, race/ethnicity, smoking status, and self-reported health status.

c Significantly different from MVPA nonbout at α = .05.

d Significant at α = .05.

**Table 4:** Reduction in BMI and Waist Circumference by Bout^a^ and Nonbout MVPA ACC Counts^b^ Per Day Among Selected Participants From the National Health and Nutrition Examination Survey, 2003-2004^c^

Category	**Total**		**Men**		**Women**	

	**β Coefficient**	***P *Value**	**β Coefficient**	***P *Value**	**β Coefficient**	***P *Value**
**BMI**						
MVPA bout ACC counts/day	−0.15	<.001^e^	−0.09	.001^e^	−0.27^d^	<.001^e^
MVPA nonbout ACC counts/day	−0.07	.005^e^	−0.08	.001^e^	−0.06	.16
*R* ^2^	0.09		0.09		0.13	
**Waist circumference**						
MVPA bout ACC counts/day	−0.36	<.001^e^	−0.24	.001^e^	−0.57^d^	<.001^e^
MVPA nonbout ACC counts/day	−0.23	<.001^e^	−0.24	<.001^e^	−0.23	.07
*R* ^2^	0.19		0.18		0.16	

Abbreviations: BMI, body mass index; MVPA, moderate- to vigorous-intensity physical activity; ACC, accelerometer.

a At least 10 consecutive minutes of MVPA.

b ACC count was derived by using physical activity measurements from the Actigraph AM-7164 activity monitor (ActiGraph LLC, Fort Walton Beach, Florida). Participants wore the monitor, which used an internal time clock and extended memory to record and store the magnitude of acceleration and deceleration associated with movement. The recorded signal was then amplified and filtered, and the resulting signal was scored as an ACC count. We coded 1 minute of ACC count data as 1 minute of MVPA if the ACC count was ≥760 (16). Activity counts were divided by 10,000 for these calculations.

c Total and sex-specific models adjusted for age, age-squared, race/ethnicity, smoking status, and self-reported health status.

d Significantly different from MVPA nonbout at α = .05.

e Significant at α = .05.
